# Unveiling Advances in Membrane Materials for CO_2_ Separation and Direct Air Capture (DAC): From Membrane Design to Applications

**DOI:** 10.1021/acsami.6c01406

**Published:** 2026-03-19

**Authors:** Guoqiang Li, Jakub Zdarta, Teofil Jesionowski, Agata Zdarta

**Affiliations:** Institute of Chemical Technology and Engineering, Faculty of Chemical Technology, 49632 Poznan University of Technology, Berdychowo 4, 60965 Poznan, Poland

**Keywords:** direct air capture, membranes, gas separation, carbon dioxide, metal organic frameworks (MOFs), covalent organic frameworks (COFs)

## Abstract

The increase in carbon dioxide (CO_2_) concentration in the atmosphere has resulted in adverse and irreversible effects in terms of climate change and global warming. To limit the temperature rise to less than 2 °C by the end of this century, it is urgent to reduce the CO_2_ concentration in the atmosphere. Direct air capture (DAC) is considered a carbon-negative emission technology which could efficiently remove CO_2_ from air. Membrane gas separation is a promising technology for CO_2_ capture, owing to its higher energy efficiency, greater scale-up ability, and smaller carbon footprints compared with conventional sorption processes. The application of membranes in the DAC process (m-DAC) is still in its infancy, owing to the low CO_2_ concentration (400 ppm) in air. However, simulations and laboratory studies have demonstrated the feasibility of m-DAC. With the development of high-performance membrane materials and the design of multistage membrane processes, the implementation of m-DAC will be a promising strategy for the efficient reduction of CO_2_ concentration in air. This review presents current studies on the m-DAC process and recently developed membranes for CO_2_/N_2_ separation which could be potentially used in that process, as well as highlighting research gaps that currently represent obstacles to the wider use of membranes for m-DAC. In conclusion, challenges and future prospects are presented, along with a roadmap for the future development of m-DAC, to provide a deeper insight into m-DAC processes.

## Graphical abstract

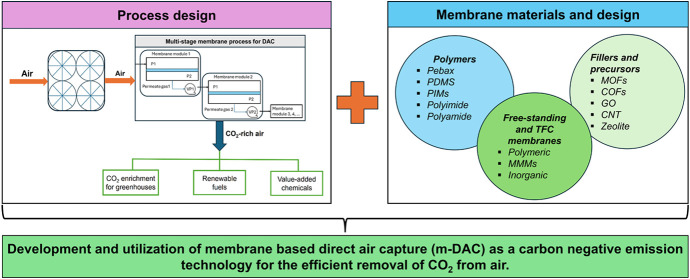

## 1 Introduction

The emission of carbon dioxide (CO_2_) and the increase in its concentration in the atmosphere have produced adverse and irreversible effects in terms of climate change and global warming. The dangers of global warming have been clearly demonstrated by catastrophic environmental problems such as rising sea levels, melting of glaciers, droughts, floods, devastating wildfires, and intense tropical cyclone activity.^1^ The atmospheric CO_2_ concentration in urban and industrial areas is generally higher than the average value of 400 ppm. For instance, in the urban area of Shanghai, the CO_2_ concentrations in roadside areas, residential areas, and green spaces are 453, 436, and 429 ppm, respectively.^2^ The CO_2_ concentration at industrial sites is about 470 ppm.^3^ Therefore, it is urgent to reduce the CO_2_ concentration in the atmosphere to meet the target of limiting global warming to 1.5 °C or below 2 °C by the year 2100, set by the International Panel on Climate Change (IPCC).^4^ To meet the 1.5 °C target, over 100 gigatons of greenhouse gas, mainly CO_2_, will have to be captured from the atmosphere annually by 2050.^5^ The reduction of CO_2_ emissions alone is not sufficient to achieve the 1.5 °C target. The carbon capture and sequestration (CCS) process is used to capture CO_2_ at specific points; for example, from the flue gas generated in energy installations and in cement and steel production.^6^ To achieve net negative CO_2_ emissions, direct air capture (DAC) is considered a more attractive option and a complementary technology for the CCS process. Compared with other CO_2_ removal technologies such as afforestation and reforestation, and bioenergy with carbon capture and storage, DAC requires much less land and water consumption.^7^ Compared with CCS processes, which can take place in limited locations, DAC can be implemented in any location, ideally close to storage and utilization sites.^8^ Therefore, DAC is considered a promising negative CO_2_ emission technology.

Conventionally, absorption and adsorption technologies are utilized in DAC processes.^9^ In sorption-based DAC processes, desorption is needed to regenerate the liquid or solid sorbents and release the captured CO_2_ for storage or further processing. The regeneration of sorbents requires high energy consumption. In addition to the high energy consumption in scrubbing DAC processes, this process has a larger water and land footprint. In adsorption DAC processes, the coadsorption of water may lower the adsorption capacity of solid porous sorbents, while the solid porous sorbent might be thermally degraded in the repeated adsorption–desorption cycles.^1^ Although sorption-based DAC processes have demonstrated high CO_2_ capture performance, the above-mentioned drawbacks limit their further large-scale application.

The low CO_2_ concentration (400 ppm) in air results in challenges for membrane-based DAC processes (m-DAC), due to the low driving force for the transport of CO_2_ through membranes. However, these challenges can be overcome by the proper selection of membranes with high gas permeance (unit: GPU; 1 GPU = 3.35 × 10^–10^ mol m^–2^ s^–1^ Pa^–1^), gas permeability (unit: Barrer; 1 Barrer = 3.35 × 10^–16^ mol m m^–2^ s^–1^ Pa^–1^) and selectivity, the design of a multistep membrane separation process, and the optimization of the operational parameters.^10^ The application of membrane technology in DAC processes is considered a promising and more effective approach for direct CO_2_ capture from air,^11^ owing to advantages such as high energy efficiency, simple operation, high scale-up ability, high packing density and modularity, and small carbon footprint.^9^ In contrast to sorption-based DAC processes, m-DAC requires neither chemicals such as liquid sorbents, nor high energy consumption for the release of captured CO_2_ and regeneration of sorbents.^1^ Moreover, m-DAC processes can be run in continuous separation mode, and the captured CO_2_ can be directly collected on the permeate side. With the development of high-performance membranes with high gas permeance and selectivity, the medium and small size of membrane separation devices means that they can be designed and employed at various scales and locations, including indoor environments such as office buildings and shopping malls, which may lead to ubiquitous CO_2_ capture from air.^12^

Most publications focus on the fabrication and application of membranes in CO_2_ separation from point sources, such as CO_2_ separation from flue gas and natural gas.^13^ The literature on the application of membranes in DAC processes is limited. Some studies focus on sorption-based DAC processes,^14^ while some analyze DAC processes from technical, commercial, economic, and environmental standpoints.^9^ Therefore, there is a strong need for a review of recent studies focusing on the application of membranes in DAC processes. The main goal of this review is to highlight the potential and discuss the feasibility of the application of membranes in DAC processes. Emphasis is placed on the importance and advantages of m-DAC processes in achieving negative CO_2_ emissions. An analysis of the feasibility and the process parameters of membranes in DAC processes is provided, followed by a comprehensive summary of the development in the past five years of advanced membranes with high CO_2_/N_2_ separation performance, including polymer-based membranes, mixed matrix membranes, and inorganic membranes, with indication of their potential applications in m-DAC processes. Some examples of applications of membranes in DAC processes on a laboratory scale are also presented and discussed. At the end of the review, challenges and future prospects are described, to give a picture of future directions in the design and fabrication of high-performance membranes for m-DAC processes.

## 2 Literature Retrieval

A search of peer-reviewed papers was conducted to study the current status and identify gaps in research on membrane-based direct air capture (m-DAC) processes. The search resources used were Science Direct, Scopus, and Google Scholar. The main search keywords were “direct air capture”, “membrane technology”, “polymeric membranes”, “mixed matrix membranes”, “inorganic membranes”, and “CO_2_ separation”, and the time frame of the search was from 2020 to 2025. As shown in Figure 1, there is growing research interest in m-DAC processes and the preparation of various types of membranes for CO_2_ separation. Following a careful analysis of titles, keywords, and abstracts, 108 papers were found to be very closely related to the topic of interest and were selected as references for the preparation of this review.

**Figure 1:**
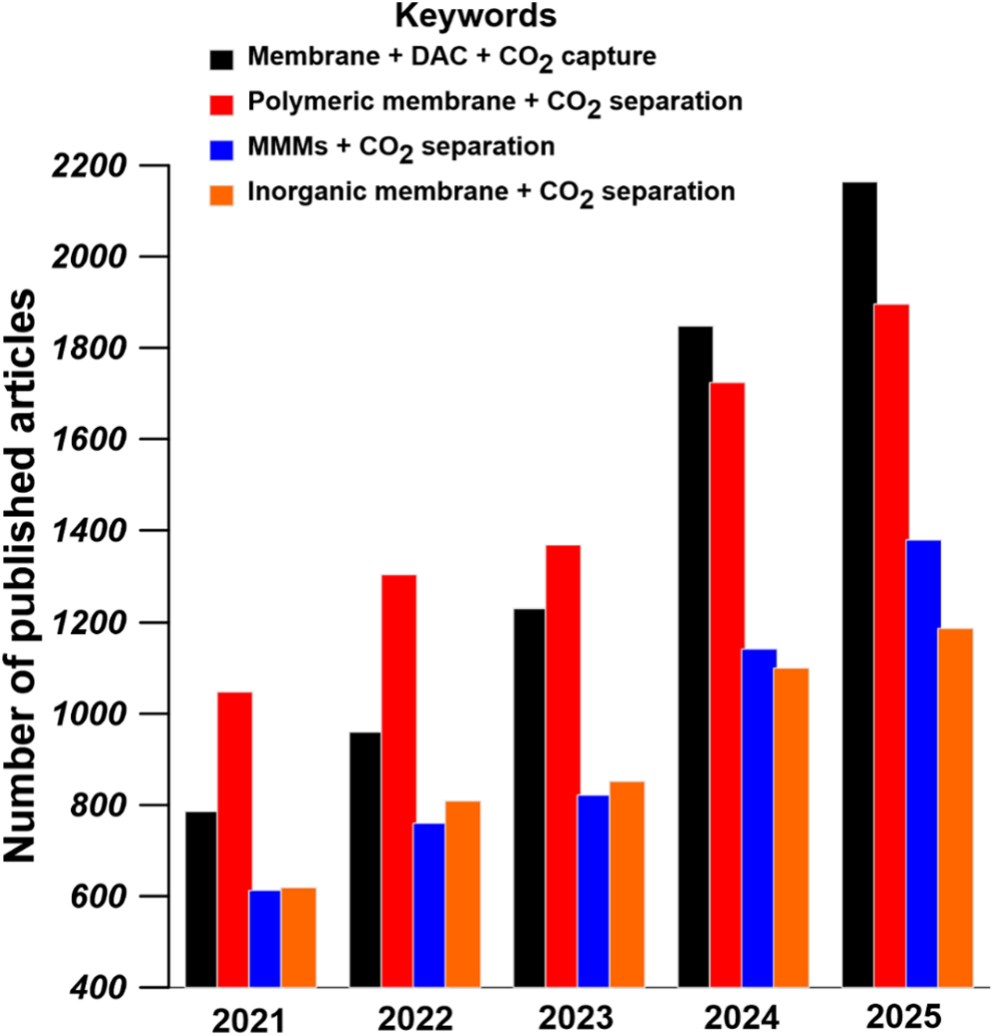
Science Direct statistics for published articles related to the review topic, obtained by searching for the keywords “direct air capture”, “membrane technology”, “polymeric membranes”, “mixed matrix membranes”, “inorganic membranes”, and “CO_2_ separation” within the 2021–2025 period (based on data as at 1 October 2025).

## 3 Overview of Direct Air Capture (DAC) Technologies

Depending on the CO_2_ emission source and process design, various carbon capture technologies are applied for CO_2_ capture, storage and utilization, including precombustion carbon capture, postcombustion carbon capture, and DAC processes.^15^ More details related to carbon capture technologies in general can be found elsewhere.^15,16^ This work focuses on m-DAC processes. Therefore, the main DAC technologies, such as liquid scrubbing, solid sorbent, electrochemical, membrane, and cryogenic, are summarized in this section to provide a comprehensive picture of these methods. The advantages and drawbacks of the particular DAC technologies are presented and compared in Table 1.

**Table 1:** Summary and Comparison of Various DAC Technologies

DAC technology	advantages	drawbacks	refs
Hydroxide–carbonate DAC	Relatively fast adsorption kinetics.	High generation temperatures.	^17^
	Chemically stable, low volatility, and low toxicity of hydroxide solvents.	High carbon footprint.	
		High capital investment.	
	Suitable for large-scale operations.	Water losses and scaling on equipment.	
Amine-scrubbing	Lower regeneration temperatures compared with hydroxide solvents.	High water and land footprint and high regeneration energy.	^18^
	High availability, low costs, and strong binding forces.	High volatility and toxicity.	
		Corrosiveness and fouling of equipment.	
Temperature–vacuum swing adsorption (TVSA)	Compared with liquid scrubbing approaches, TVSA requires a lower capital cost, lower temperatures for regeneration, and higher capture capacities.	Co-adsorption of water resulting in lower adsorption capacity and higher energy for regeneration.	^19^
		Low CO_2_ selectivity, low sorption kinetics, and high pressure drops.	
Moisture swing adsorption	Lower energy than with other thermal-based DAC approaches.	High sensitivity to local weather conditions.	^20^
	A heat pump cycle for heat recovery.	Requirement for large heat exchanger areas resulting in high capital cost.	
Electrodialysis DAC	High CO_2_ purity in permeate.	Utilization of expensive membranes	^21^
	Possibility of combining multiple cells in series, leading to lower voltages and lower energy requirements.	that increase the overall capture cost.	
		The release of gaseous CO_2_ leads to bubble formation within cells.	
Membrane DAC	Smaller footprint, simpler operation, and high scalability.	Vacuum applied on permeate side results in increased energy consumption.	^10^
	No need to use high-grade heat to regenerate sorbent and separate CO_2_.	Strong parametric sensitivity between membrane permeability and selectivity.	
	Continuous separation mode.		
Cryogenic DAC	Recovered CO_2_ has very high purity.	Very high energy requirement and highly energy-intensive process.	^22^
	High CO_2_ recovery rates.	High sensitivity to moisture in air.	
	Production of CO_2_ in a liquid state suitable for transportation and storage.		

## 4 Considerations for Membrane-Based Direct Air Capture (m-DAC)

### 4.1 Discussion on the Feasibility of m-DAC

Membrane technology has been used in the separation of CO_2_ from flue gas containing 10–30% CO_2_ via the carbon capture sequestration (CCS) process, owing to its ease of scale-up, reduced chemical use, operational simplicity and flexibility, and low energy consumption.^23^ Compared with applications of membranes in the separation of CO_2_ from flue gas, their utilization in the DAC process is more challenging due to the low CO_2_ concentration (400 ppm).^24^ The low concentration of CO_2_ in air means that higher energy consumption is required to achieve sufficient driving force via compression on the feed side or vacuuming on the permeate side for CO_2_ transport through membranes, and to meet the targets for CO_2_ purity (>95%) and capture rate (>90%) for geological storage.^12^ However, with the development of membranes with high CO_2_ permeance and selectivity, and processes utilizing low CO_2_ concentration, the m-DAC process has demonstrated high feasibility and potential for the reduction of CO_2_ in air.^16,25^ What is more, a simulation study from Fujikawa has proven the feasibility of m-DAC.^26^ In that work, the membrane applied was assumed to have gas permeance of 40,000 GPU with a CO_2_/N_2_ selectivity of 70. The pressure on the feed side was atmospheric pressure, while that on the permeate side was 5 kPa, obtained by vacuuming. A four-stage separation process was applied. It was found that CO_2_ concentrations of 10% and 40% were achieved in the third and fourth separation stages, respectively. The required membrane area was 3.2 m^2^ kg^–1^ CO_2_ per day, and the volume of the membrane module was less than 0.01 m^3^. The amount of CO_2_ from the energy consumed in capturing 1 kg CO_2_ from the air was 0.6 kg, indicating net negative CO_2_ emission.

Even though the average CO_2_ concentration in the air is as low as 400 ppm, CO_2_ concentrations in the indoor human environment, such as offices, schools, shopping malls, and places where people gather, are much higher. For instance, the average CO_2_ concentration in classrooms is in the range 750–2000 ppm.^27^ The average CO_2_ concentration in office buildings is between 516 and 1300 ppm.^28^ These indoor places in cities and urban areas are desirable candidate sites for CO_2_ capture via the m-DAC process, since the higher CO_2_ concentration on the feed side in such places may significantly lower the energy consumption and increase CO_2_ purity in the permeate.^12^ For example, the mass of CO_2_ in the exhausted air is about 17,000 tons/year in a building with an office area of 179,013 m^2^ and space of 537,039 m^3^.^29^ Hence, if small-scale m-DAC units were installed in buildings in all cities, millions of tons of CO_2_ could be removed from the air. The design and construction of green and smart cities are aimed at the improvement of energy efficiency, the reduction of carbon emissions, and the enhancement of sustainability.^30^ The integration of DAC processes in the construction of green and smart cities may further accelerate carbon emission reduction and the green transformation of production and lifestyle, and achieve a clean and low-carbon economy and environment.^30^

Although the purity of the captured CO_2_ from the m-DAC process is not as high as 90%, the low-concentration CO_2_ product can be directly used for CO_2_ enrichment or supplementation in greenhouses to improve the photosynthetic rates and yields of plants.^16^ In such applications, the required CO_2_ concentration can feasibly be obtained using a single-stage m-DAC process, which can continuously supply CO_2_-enriched air to indoor agricultural facilities. Besides the direct use of CO_2_ collected from the m-DAC process, CO_2_ can be converted into value-added products such as methane, methanol, and commodity chemicals.^31^ High-purity CO_2_ is preferred in the CO_2_ conversion reaction since it can enhance the reaction efficiency. However, the production of high-purity CO_2_ requires more energy. Therefore, the utilization of low-concentration CO_2_ captured from the m-DAC process is necessary and cost-effective.^32^

### 4.2 Effect of Process Parameters on m-DAC

Both the structure of the membrane and its performance and process parameters play key roles in the membrane separation process. Therefore, this section discusses the effects of gas permeance, selectivity, stage cut, and pressure ratio on the overall performance of the m-DAC process. Membranes considered for m-DAC should have a gas permeance higher than 10,000 GPU, owing to the low CO_2_ concentration in air.^26^ Moreover, a membrane with high gas permeance is desirable for the design of multistage separation processes with a reasonable module size.^10^ As shown in Figure 2a, a three-stage membrane separation process may significantly increase the CO_2_ concentration in the permeate (Figure 2b) and reduce the energy consumed in capturing 1 kg CO_2_ via m-DAC (Figure 2c). A single-stage membrane separation process can barely achieve a 1% CO_2_ concentration in the permeate, even when using membranes with CO_2_ selectivity above 70. Therefore, the design of a multistage membrane separation process is necessary for the direct capture of CO_2_ from air. The use of membranes with high selectivity for CO_2_ over other gases in air (N_2_, O_2_, and Ar) is crucial to improve the overall performance of the membrane separation process. In a three-stage separation process, the CO_2_ concentration in the permeate increased significantly from 3% to 20% when the CO_2_ selectivity of the membranes was raised from 10 to 30. The improvement of CO purity in the permeate becomes moderate when the CO_2_ selectivity is more than 30 (Figure 2c). Similarly, the energy consumed in capturing 1 kg CO_2_ decreased significantly when the CO_2_ selectivity of the membranes was increased from 10 to 30. The decrease in energy consumption becomes weaker when the CO_2_ selectivity is more than 30 (Figure 2c). Therefore, to achieve a realistic membrane module size and reasonable energy consumption, the CO_2_ selectivity of membranes used for the m-DAC process should be at least 30. Membranes with high gas permeance and selectivity are preferred for use in the m-DAC process. The preparation and modification of high-performance membranes with high operational stability are discussed in Section 5.

**Figure 2:**
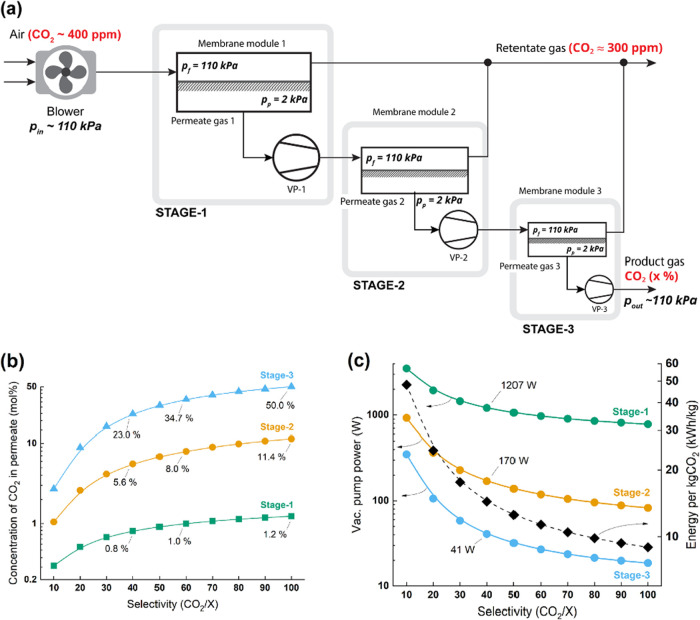
(a) Scheme of a three-stage membrane process for CO_2_ separation from air. (b) Dependence of CO_2_ purity in the product stream on membrane selectivity. (c) Requirements for the vacuum pump power (circles) and total daily energy/per kg of CO_2_ (diamonds) depending on membrane selectivity (pressure ratio setting: φ = 50).^26^ Copyright 2020 Springer Nature. CC BY 4.0 License.

The pressure ratio φ refers to the ratio of the total pressure on the feed side to the total pressure on the permeate side, which limits the mole fraction of CO_2_ on the permeate side. As shown in Figure 3a, the higher the pressure ratio, the greater the resulting purity of CO_2_ in the permeate. The increment of CO_2_ concentration in the permeate slows down when the pressure ratio is higher than 30. Therefore, a high pressure ratio of more than 30 is needed in the m-DAC process to provide sufficient driving force for the transport of CO_2_ through membranes and to ensure CO_2_ purity in the permeate. What is more, the use of a vacuum on the permeate side to achieve a sufficient pressure ratio requires much less energy consumption than feed compression.^16^ The stage cut (θ) refers to the ratio of permeate flow and feed flow. When the stage cut increases, the CO_2_ concentration in the permeate significantly decreases (Figure 3c), while the energy consumption and recovery increase (Figure 3d). The stage cut, CO_2_ purity and recovery should be finely controlled in the m-DAC process, depending on the final application of the captured CO_2_.^25^ In work by Castel et al., a low stage cut was preferred owing to the need to guarantee the purity of the CO_2_ and the lack of fixed recovery.^10^

**Figure 3:**
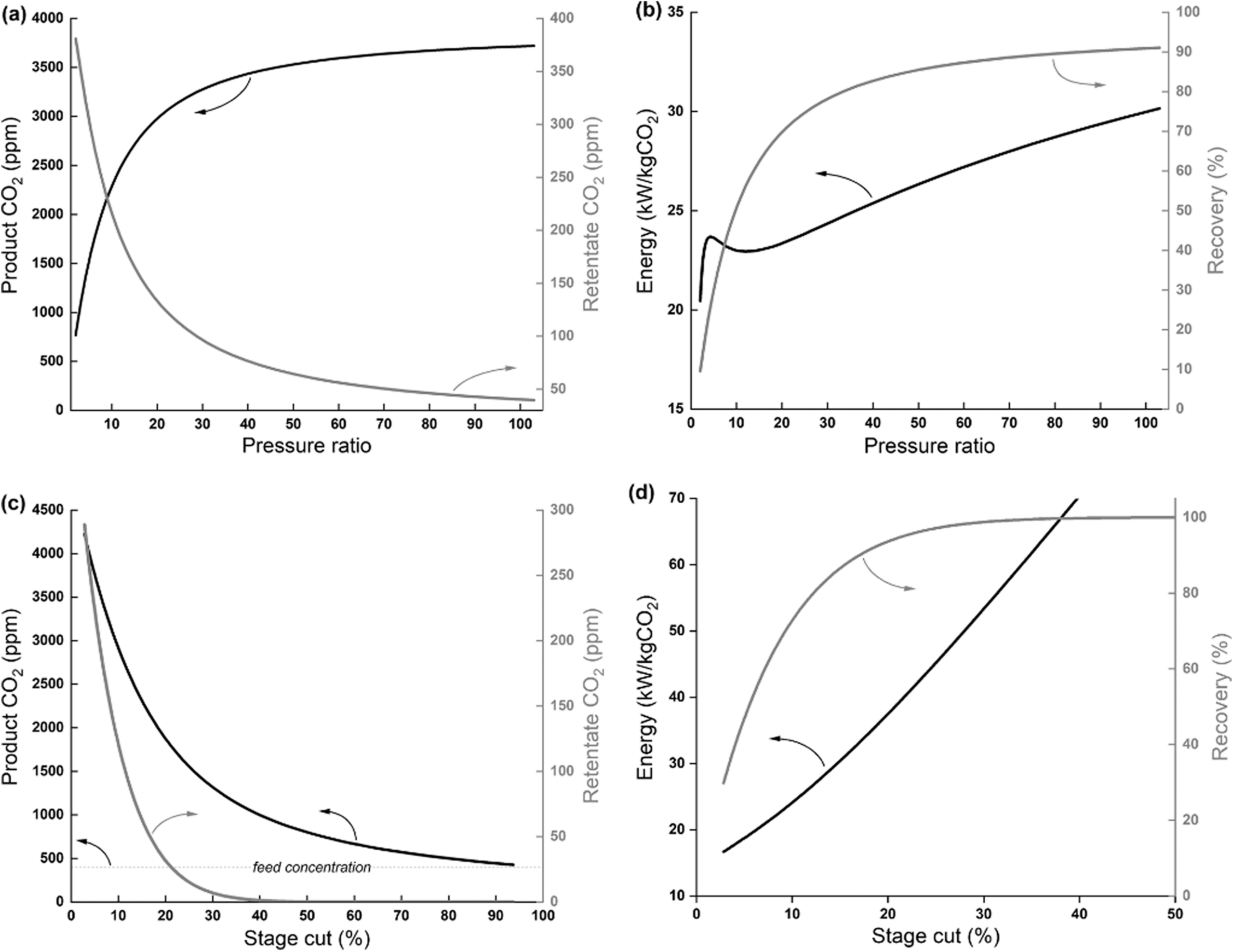
Influence of the pressure ratio φ and stage cut (θ) on CO_2_ separation from the air using a CO_2_-selective membrane. (a) Dependence of CO_2_ concentration in permeate and retentate streams and (b) CO_2_-mass normalized daily energy requirements on the pressure ratio (other parameters: stage cut θ ∼ 5–10; selectivity CO_2_/X = 30, where X denotes other components of air: N_2_, O_2_, Ar). (c) Dependence of CO_2_ concentration in permeate and retentate streams and (d) CO_2_-mass normalized daily energy requirements on stage cut (other parameters: pressure ratio φ = 20; selectivity CO_2_/X = 30, where X denotes other components of air: N_2_, O_2_, Ar).^26^ Copyright 2020 Springer Nature. CC BY 4.0 License.

### 4.3 Energy Consumption and Techno-Economic Analysis

The main energy consumption in the m-DAC process is for the generation of driving force for the movement of massive volumes of ambient air through the membrane modules. For example, the minimum energy consumed by the current commercial membrane Polaris (2,000 GPU, CO_2_/N_2_ = 30) for DAC is 18,000 Kwh/ton CO_2_. Membranes with much higher CO_2_ permeance (10,000–40,000 GPU) can lower the energy consumption to 12,000 Kwh/ton CO_2_.^16^ A highly selective high-performance membrane (2,500 GPU and CO_2_/N_2_ = 680) can significantly lower the energy consumption to 3000 Kwh/ton CO_2_ using only permeate vacuuming.^10^ It may be found that membrane materials have large impacts on energy consumption in m-DAC processes. There is an urgent need for the development of advanced membrane materials with high gas permeance and selectivity.

According to Fujikawa’s and Castel’s simulation studies^10,26^ and the above discussion on process parameters (Section 4.2), m-DAC offers high technological feasibility under the following conditions. (1) Membrane materials should possess very high CO_2_ permeance and desirable selectivity of CO_2_/X (X refers to N_2_, O_2_, and Ar). For example, a CO_2_ permeance of 10,000 GPU is preferred to achieve a realistic membrane module size less than 15.6 m^3^ (2.5 m × 2.5 m × 2.5 m; less than 5000 m^2^ membrane area per ton CO_2_ captured per day in such a module size, based on plate and frame modules with a membrane area to volume ratio of 320–490 m^2^/m^3^). The high selectivity of membranes results in higher CO_2_ purity and lower energy consumption (Figure 2). (2) Multistage continuous operation is necessary to obtain a higher-purity CO_2_ product at lower energy consumption. (3) A higher pressure ratio of more than 30 is necessary to obtain sufficient transmembrane CO_2_ driving force. (4) Permeate vacuuming is preferred over feed compression to obtain transmembrane CO_2_ partial pressure owing to its significantly lower energy consumption.

For the purposes of a techno-economic assessment, it was reported that the cumulative cost of membrane separation in a DAC process ranged from $91 to $95/tCO_2_, suggesting that in combination with lower oil prices and a lower enhanced oil recovery (EOR), factors may be commercially available with proper policy supports.^33^ The cost estimates for DAC are subject to large uncertainties and depend on the process boundary considered and the approach to economic assessment, especially for large-scale utilization of DAC.^9^ Fan et al.^9^ evaluated a DAC with utilization (DAC-U) process by using life cycle assessment (LCA) from environmental and economic standpoints. It was found that although the DAC-U represents a carbon negative capable technology with positive lifestyle and environmental outcomes, high capital costs present a significant barrier to deployment. To overcome this barrier, robust policy support including subsidies or fuel credits may be necessary. Further technological innovation and efficiency gains will also close this gap.^9^

Various membrane module types (spiral wound, hollow fiber, plate and frame, or other) may be used for carbon capture, but they need to be designed to optimize low pressure vacuum operation.^34^ A hollow fiber module might further decrease the membrane module size owing to its higher surface area-to-volume ratio, compactness and modality, further improving the feasibility of m-DAC processes in large-scale applications. Moreover, hollow fiber membranes can be designed with a membrane contactor, allowing the utilization of sorption mechanisms for CO_2_ capture.^35^

## 5 Membrane Materials for CO_2_ Separation and m-DAC Applications

Membrane materials play a pivotal role in membrane separation processes for CO_2_ capture. Various types of polymers and inorganic materials have been developed and utilized in the preparation of membranes for CO_2_ capture.^25^ Rubbery polymers such as poly­(dimethylsiloxane) (PDMS)^36,37^ and poly­(ethylene oxide) (PEO), followed by polymer-based products such as Pebax,^38,39^ glassy polymers such as polyimide (PI),^40^ and polymers of intrinsic microporosity (PIMs),^41,42^ have been used for the preparation of free-standing and thin film composite membranes. Metal organic frameworks (MOFs),^43,44^ covalent organic frameworks (COFs),^45,46^ and 2D materials such as graphene oxide (GO) and MXenes^47,48^ have been used for the fabrication of supported inorganic membranes or applied as nanofillers in the preparation of mixed matrix membranes (MMMs).^49^ Each type of material features inherently unique microstructure and physicochemical properties, which result in different CO_2_ transport pathways. For instance, the high free volume of membranes results in a higher diffusion rate for CO_2_ molecules. A smaller membrane pore size allows the fast transport of smaller CO_2_ molecules while inhibiting the transport of larger molecules such as N_2_. Functional groups in membranes, such as amino and carboxyl groups, can enhance the solubility and facilitate the transport of CO_2_ molecules, owing to their interaction with such groups. Therefore, membranes with high CO_2_ permeance can be designed and fabricated by tuning the intrinsic microstructure and properties of polymers. In this section, recent developments involving polymeric membranes, inorganic membranes, and MMMs with high CO_2_ permeability or permeance and CO_2_/N_2_ selectivity will be systematically summarized and discussed. Most of the membranes presented in Section 5 were tested under flue gas conditions and compositions. They exhibited very high CO_2_ permeance or permeability and desirable CO_2_/N_2_ selectivity, demonstrating high potential for their applications in m-DAC processes. Although these membranes have mainly been developed and applied for CO_2_ capture from point sources such as flue gas, they are desirable candidates for use in m-DAC processes, owing to their high CO_2_ permeability or permeance and CO_2_/N_2_ selectivity.

### 5.1 Polymeric Membranes

Polymeric membranes are the most studied and commercially available membranes for CO_2_ separation, owing to their low production cost, high processability, and ease of scale-up.^50,51^ Table 2 summarizes the fabrication techniques, testing conditions, and CO_2_ separation performance of recently developed polymeric membranes made from rubbery and glassy polymers. Polymeric membranes are generally fabricated as free-standing membranes using the solution casting technique,^42^ and as supported thin film composite (TFC) membranes using the coating technique^37,52^ and interfacial polymerization.^53^

**Table 2:** Representative Examples of Recently Developed (in the Last Five Years) Polymeric Membranes for CO_2_/N_2_ Separation (1 GPU = 3.35 × 10^–10^ mol m^–2^ s^–1^ Pa^–1^, 1 Barrer = 3.35 × 10^–16^ mol m m^–2^ s^–1^ Pa^–1^)

membrane materials	fabrication method	operating conditions	CO_2_/N_2_ mixture	CO_2_ permeability/permeance	CO_2_/N_2_ selectivity	long-term performance	refs
Polyimide 6FDA-TMPD	Dip coating	30 °C, 1.3 bar	15/85 vol %	99.7 GPU	24.8		^52^
			Single gas	645 Barrer	20.6		
Polyimides 6FDA-APHBA/DAM	Solution casting	35 °C, 3.45 bar	Single gas	500.9 Barrer	21.4	After 60 days, CO_2_ permeability decreased by 23.6%, while CO_2_/N_2_ selectivity increased to 24	^40^
Oxygen-rich polyimide/PDMS/PEI	Interfacial polymerization	25 °C, 2 bar	20/80 vol %	150 GPU	42.9		^53^
PDMS/Matrimid	Coating	25 °C, 21.5 bar	Single gas	1099 GPU	52.2	Stable performance for 11 days	^37^
Adamantane-grafted PIM-1 (AOPIM-1)	Solution casting	35 °C, 1 bar	Single gas	2483.6 Barrer	31.2		^42^
Amine-functionalized PIM-1 (PIM-1-NH_2_)	Solution casting	35 °C, 1 atm	Single gas	2300 Barrer	20		^58^
PIM-1@Noria-based porous organic polymer	Solution casting	30 °C, 1 bar	15/85 vol %	7131 Barrer	22.1	After 180 days, CO_2_ permeability decreased by 33.4%	^41^
PIM-EN-40	Solution casting	35 °C, 2 bar	Single gas	11,512 Barrer	21.8	After 193 days, CO_2_ permeability decreased by 30.5%	^59^
Pebax 1657/PSf	Spray-coating	25 °C, 1 bar	20/80 vol %	260 GPU	51	Stable performance for 100 h consecutive test	^38^
r-Pebax 1657/PTMSP/PSf	Bar coating	25 °C, 5 bar	50/50 vol %	2371 GPU	45	14% decrease in CO_2_ permeance during 50 h consecutive test	^56^
Pebax 3533/PSf	Phase inversion	35 °C, 3 bar	15/85 vol %	127 GPU	21.4		^39^
PPPS/PDMS/PSf	Dip coating	25 °C, 5 bar	15/85 vol %	1050 GPU	64	Stable performance for 200 h consecutive test	^61^
PPPS/TMC-PDMS/PSf				785 GPU	78		
HPEO/PDMS/PSf	Dip coating	35 °C, 2 atm	15/85 vol %	850 GPU	37	Stable performance for 100 h consecutive test	^62^
PDMS/PES	Dip coating	25 °C, 20 bar	15/85 vol %	1853.8 GPU	13.1	CO_2_ permeance and CO_2_/N_2_ selectivity increase in the first 3 days, then remain stable for the next 7 days	^36^
PolyActive/PDMS/PES	Dip coating	25 °C, 2 bar	15/85 vol %	262 GPU	24.3	Stable separation performance over 40 days	^63^
PolyActive		21 °C, 3 bar		1481 GPU	60		^6^
Polaris gen1 (commercially available)				1000 GPU	50		^64^
Polaris gen2 (commercially available)				1700 GPU	≈50		^34^
Polaris gen3 (commercially available)				3000 GPU	≈50		^34^

Rubbery polymers such as PEO-containing block copolymers (Pebax) demonstrate high CO_2_ solubility selectivity owing to their high content of ether oxygen.^54^ The ether linkage has a high affinity to CO_2_ molecules via the dipole–quadrupole interaction.^55^ The interaction between ether groups and CO_2_ molecules increases the solvation of CO_2_ and CO_2_ solubility. Jiang et al.^38^ fabricated a Pebax 1657 thin film membrane on a porous polysulfone (PSf) substrate using a direct spray-coating technique. Subsequently, the PDMS sealing coating was applied to seal defects on the membrane surface. The prepared PDMS/Pebax 1657/PSf TFC membranes exhibited high CO_2_ permeance equal to 260 GPU and a CO_2_/N_2_ selectivity equal to 51 in tests using a CO_2_/N_2_ (20/80 vol %) gas mixture. It was found that the high CO_2_ permeance of the prepared membranes was related to the formation of an ultrathin selective layer with a thickness less than 100 nm, and the inhibited polymer intrusion. Park et al.^56^ prepared defect-free, ultrathin, and CO_2_-selective TFC membranes using a transient-filler (TF) treatment, with poly­(ethylene glycol) (PEG) as TF and Pebax 1657 as matrix, and the subsequent removal of PEG. As shown in Figure 4, the gas permeable poly­[1-(trimethylsilyl-1-propyne)] (PTMSP) gutter layer was first coated on the PSf porous substrate to prevent the intrusion of PEG/Pebax 1657 solution. Subsequently, the PEG/Pebax 1657 layer was coated on the PTMSP surface, followed by PEG removal treatment to form the final r-Pebax 1657 selective layer. The prepared membranes exhibited a high CO_2_ permeance of 2,371 GPU along with a CO_2_/N_2_ selectivity of 45, resulting from the extremely low membrane thickness of 96 nm, the additional gas diffusion channels from the increased fractional free volume, and the reduced crystallinity of the selective layer. As the above studies show, TFC-based membranes have demonstrated high CO_2_ permeance. High CO_2_ permeance membranes with high or moderate CO_2_ selectivity are critical for efficient CO_2_ capture processes. The above examples of TFC membranes demonstrate that reducing the membrane thickness to a few hundred nanometers may significantly increase the CO_2_ permeance. However, the development of novel coating techniques is crucial for the formation of defect-free TFC membranes with high CO_2_ separation performance.

**Figure 4:**
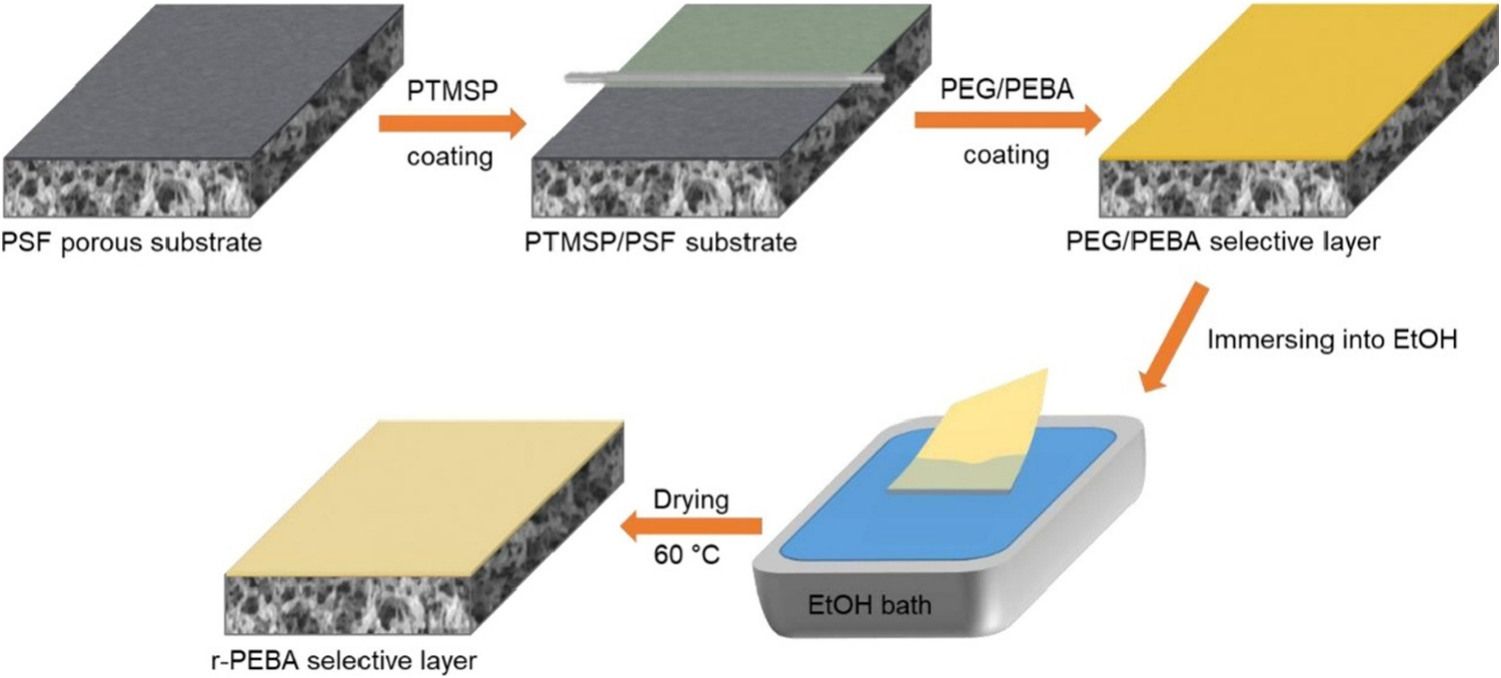
Schematic illustration of the fabrication of r-PEBA TFC membranes.^56^ Reprinted with permission from Elsevier. Copyright 2023.

Glassy polymeric membranes generally contain stiff polymer chains and have stronger size-sieving ability.^53^ The diffusion of gas molecules in such membranes mainly depends on the size of those molecules. Therefore, glassy polymeric membranes exhibit high CO_2_ selectivity but low permeability. Plasticization and physical aging commonly occur in glassy polymeric membranes, limiting their CO_2_ separation performance.^50^ Therefore, resistance to plasticization and physical aging should be considered when fabricating such membranes. To develop asymmetric membranes with high CO_2_ permeance and high CO_2_/N_2_ selectivity, Ding et al.^53^ fabricated TFC membranes containing an ether oxygen-rich polyimide separation layer. The TFC polyimide membranes achieved a high separation factor equal to 429 for a CO_2_/N_2_ (20/80 vol %) gas mixture, along with a CO_2_ permeance of 150 GPU, owing to the high-polarity ether groups in the composite membrane. Moreover, the membranes displayed strong resistance to plasticization in a feed pressure range from 0.3 to 2.3 MPa.

PIMs are microporous polymers characterized by high microporosity and high free volume, resulting from the inefficient packing of rigid, highly contorted nonlinear polymer chains.^57^ PIMs have exhibited high CO_2_ permeability and moderate CO_2_/N_2_ selectivity. Therefore, as shown in Figure 5, various types of PIMs, such as ladder PIMs, PIM–PIs, and functionalized PIMs, have been designed and synthesized via copolymerization, chemical functionalization, and thermal cross-linking.^58,59^ Xu et al.^41^ synthesized Noria-based porous organic polymer and introduced it as a block into PIM-1 via copolymerization. Subsequently, the synthesized PIM-1@Noria was used to fabricate free-standing membranes by a solution casting technique. The prepared membrane exhibited a high CO_2_ permeability of 7,131 Barrer, along with a CO_2_/N_2_ selectivity of 22 for the separation of a CO_2_/N_2_ (15/85 vol %) gas mixture. The high CO_2_/N_2_ separation performance was related to the increased free volume and the enhanced CO_2_ affinity of PIM-1@Noria membranes. The prepared membrane also achieved favorable aging resistance, since the Noria block could form colloidal networks and provide rigidity to the separation membrane. Bezzu et al.^60^ fabricated an ultrapermeable PIM by introducing a rigid, bulky triptycene to the spirobisindane (SBI) unit to form a 3D contorted PIM-SBI-Trip polymer. The PIM-SBI-Trip membrane exhibited a CO_2_ permeability of 35600 Barrer and a CO_2_/N_2_ selectivity of 15.4, which is related to its strong size-sieving character and high rigidity. As the above studies show, by tuning the membrane functional groups, the fractional free volume and the crystallinity of the polymers, it is possible to increase the intrinsic gas permeability of such membranes. Moreover, the modification of polymer chains can potentially enhance the membranes’ resistance to plasticization and physical aging.

**Figure 5:**
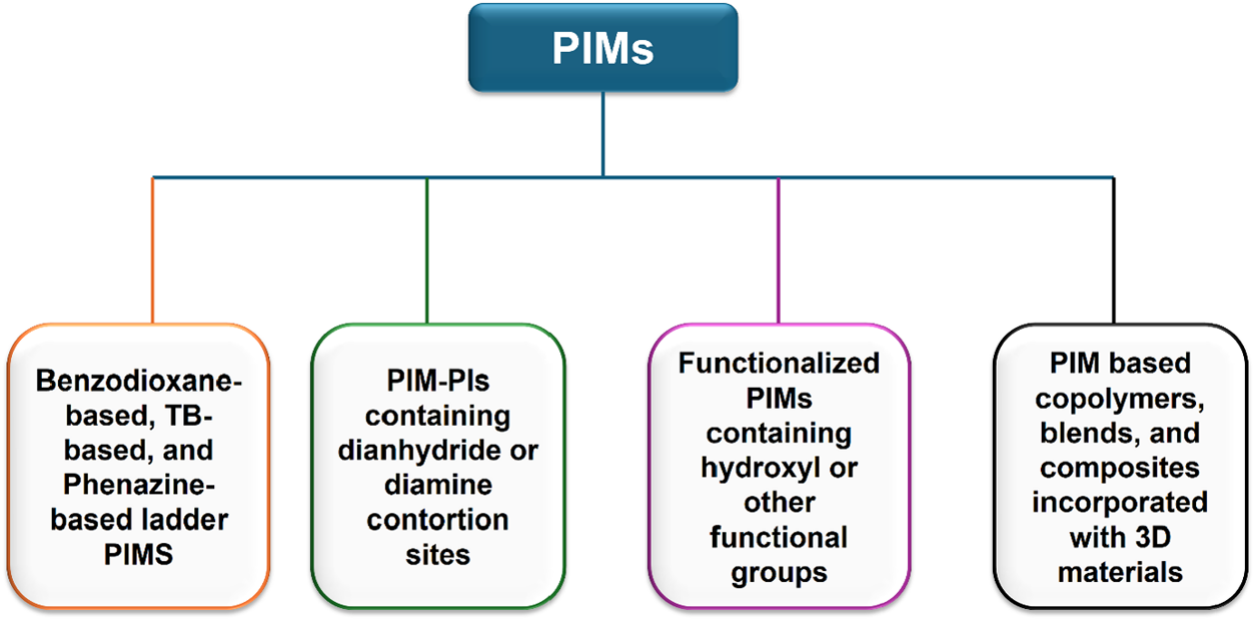
General classifications of PIMs-based membranes for CO_2_ capture.^57^ Redrawn with permission from Elsevier. Copyright 2024.

### 5.2 Mixed Matrix Membranes

Mixed matrix membranes (MMMs) consist of micro- or nanosized, porous or nonporous, inorganic or organic fillers and a polymer matrix. MMMs may significantly improve gas separation performance and avoid the permeability/selectivity trade-off encountered in the case of polymeric membranes.^65^ This is because MMMs can combine the advantages of fillers, such as high CO_2_ solubility and strong molecular sieving ability, with those of polymers, such as high processability and ease of scale-up.^49^ MMMs can be fabricated by a mixing and solution casting method^66^ or by forming the fillers in situ in the polymer matrix to ensure their homogeneous dispersion in the matrix.^67^ Recently developed MMMs containing various types of fillers are summarized in Table 3, along with their CO_2_/N_2_ separation performances. It is seen that various types of fillers have been synthesized, modified, and used in MMMs, including MOFs,^68^ COFs,^69^ graphene oxide (GO),^70^ MXene,^71^ zeolites,^72^ and carbon nanotubes (CNTs).^73^ The incorporation of fillers into MMMs can effectively change their CO_2_ solubility and diffusivity, as well as CO_2_/N_2_ solubility selectivity and diffusivity selectivity, resulting in enhanced CO_2_/N_2_ separation performance. For instance, the CO_2_ solubility increases owing to the high CO_2_ affinity of fillers such as MOFs. The CO_2_ diffusivity increases since the porous fillers can create additional pathways for gas molecules.^74,75^ The diffusivity selectivity of CO_2_/N_2_ increases owing to the enhanced molecular sieving ability of porous fillers and the tortuous gas transport pathways created by 2D nanosheets or nonporous fillers.^70^

**Table 3:** Representative Examples of Recently Developed (in the Last Five Years) Mixed Matrix Membranes (MMMs) for CO_2_/N_2_ Separation (1 GPU = 3.35 × 10^–10^ mol m^–2^ s^–1^ Pa^–1^, 1 Barrer = 3.35 × 10^–16^ mol m m^–2^ s^–1^ Pa^–1^)

membrane materials	fabrication method	operating conditions	CO_2_/N_2_ mixture	CO_2_ permeability/permeance	CO_2_/N_2_ selectivity	long-term performance	refs
1 wt % ZIF-8-PDMS/Ceramic support	Coating	25 °C, 2 bar	Single gas	4756 GPU	10.1	Stable separation performance over 14 h under high temperature 80 °C	^43^
0.8 wt % ZIF-8-PDMS/ZIF-8/Ceramic support	In situ growth Coating			3050 GPU	10.7		
10 wt % NH_2_IL-MOF-808-PIM-1	Casting	30 °C, 4 bar	50/50 vol %	10,106 Barrer	43.6	Excellent stability over a period of 72 h	^44^
8 wt % UiO-66-(CF_3_)_2_-PIM-1	Casting	60 °C, 1 bar	Single gas	5242 Barrer	33.8		^68^
8 wt % UiO-66-(CF_3_)_2_-PIM-1/PDMS/PSf	Coating		13.5/76.5 vol % + 10 vol % H_2_O	1111 GPU	43.7	Stable separation performance over 720 h	
0.7 wt % TA-LDH-PIM-1	Casting	25 °C, 1 bar	50/50 vol %	8,384 Barrer	39	After 30 days’ aging, CO_2_ permeability decreased by 20%	^66^
3 wt % [Emim][Tf_2_N]@TPB-DMTP-COF-PIM-1	Casting	30 °C, 1 bar	15/85 vol %	8477 Barrer	19.6	After 100 days’ aging, CO_2_ permeability decreased by 34%.	^45^
3 wt % PDA@TPB-TMDP-COF-PIM-1	Casting	30 °C, 1 bar	Single gas	9750 Barrer	26.4	CO_2_ permeability decreased by 16% while selectivity increased by 2.3% in 120 h consecutive test	^69^
6 wt % TpTta-COF-PIM-1	Casting	25 °C, 3 bar	15/85 vol %	9672 Barrer	26.3	Stable separation performance over 16 days	^46^
0.5 wt % MXene-PIM-1	Casting	25 °C, 3 bar	10/90 mol %	12,475 Barrer	32.7	Stable separation performance over 24 h	^71^
5 wt % PP-DETA-cPIM-1-PIM-1	Casting	25 °C, 1 bar	20/80 vol %	5699 Barrer	19.8	After 180 days’ aging, CO_2_ permeability decreased by 4.3%	^78^
15 wt % HO-UiO-66 PLs-PIM-1	Casting	30 °C, 1 bar	Single gas	7211 Barrer	26	CO_2_ permeability decreased by 12% while selectivity increased by 16% in 120 h consecutive test	^74^
10 wt % Ag + @UiO-66-NH_2_–PIM-1	Casting	25 °C, 2 bar	10/90 mol %	15,000 Barrer	30		^75^
TpPa_0.025_-PIP-TMC/PSf	Interfacial polymerization	25 °C, 1.5 bar	15/85 vol %	854 GPU	148	Stable separation performance over 70 h	^79^
0.2 wt % Cu-MOF- Pebax 3533	Casting	35 °C, 2 bar	Single gas	619 Barrer	23.9	Stable permeability and selectivity over 7 days	^76^
15 wt % 2D ZIF-8-NH_2_- Pebax 1657	Casting	35 °C, 2 bar	Single gas	460 Barrer	95		^47^
15 wt % 2D ZIF-8-NH_2_- Pebax 1657/PTMSP/PSf	Coating	35 °C, 2 bar	15/85 vol %	1250 GPU	75	Stable CO_2_ permeance and separation factor over 100 h consecutive test	
2 wt % PEG-MMT- Pebax 1074	Casting	30 °C, 1 bar	15/85 vol %	122 Barrer	178.1	Stable separation performance for 70 h	^80^
5 wt % 2D ZIF (Cu)- Pebax 2533	Casting	25 °C, 2 bar	Single gas	606 Barrer	28	Stable permeability and selectivity for 7 days	^48^
1 wt % SUM-9- Pebax 2533	Casting	25 °C, 6 bar	Single gas	539 Barrer	24.7		^81^
9 wt % mCN-Pebax 1657	Casting under magnetic field	25 °C, 2 bar	Single gas	112 Barrer	89	Stable permeability and selectivity for 192 h	^82^
5 wt % PEI@MWCNTs/Pebax 1657	Casting	25 °C, 3 bar	Single gas	150 Barrer	82		^73^
15 wt % ZnY-zeolite-nanocellulose/nylon	Vacuum filtration	25 °C, 1 bar	Single gas	266 Barrer	92.4	Stable permeability and selectivity over 160 h	^83^
2 wt % PEI@p-MMT-PVAm/PSf	Coating	25 °C, 1 bar	15/85 vol %	238 GPU	124.1	Stable separation performance over 360 h	^84^
3 wt % AGO-guar gum-chitosan	Casting	25 °C, 0.7 bar	50/50 vol %	39,734 Barrer	99.3		^70^

MOFs are commonly used fillers in MMMs for CO_2_ separation, owing to their high porosity, large surface area, molecular sieving ability, high CO_2_ affinity, tunable pore sizes and functionalities, and high compatibility with polymer matrices.^57^ Homogeneous dispersion and strong compatibility with the polymer matrix are crucial for the preparation of defect-free MMMs. To enhance the CO_2_ affinity of MOFs and their compatibility with the polymer matrix, Sun et al.^44^ modified MOF-808 with the ionic liquid [NH_2_PMIM]­[Tf_2_N] (NH_2_IL) via encapsulation and prepared NH_2_IL-MOF-808/PIM-1 MMMs for CO_2_/N_2_ separation. The prepared MMMs containing 10 wt % filler achieved the highest CO_2_ permeability of 10,106 Barrer, a CO_2_/N_2_ selectivity of 44, and long-term stability for 72 h at 30 °C and 4 bar for CO_2_/N_2_ (50/50 vol %) separation. Zhou et al.^68^ synthesized water-stable UiO-66-(CF_3_)_2_ by grafting with hydrophobic trifluoromethyl groups. It was found that the introduction of trifluoromethyl groups into UiO-66 significantly decreased its BET surface area (by 44%) and total pore volume (by 59%), but increased its hydrophobicity, as confirmed by the large water contact angle of 143°. Subsequently, PIM-UiO-66-(CF_3_)_2_/PDMS/PSf TFC MMMs were prepared by the multiple coating technique. The prepared MMMs containing 8 wt % filler exhibited stable and high CO_2_ permeance equal to 1,111 GPU and a CO_2_/N_2_ separation factor above 43 under a simulated flue gas environment, owing to the hydrophobicity, enhanced CO_2_ affinity and size-sieving ability of the UiO-66-(CF_3_)_2_ filler. Besides 3D MOFs, 2D MOF nanosheets have also demonstrated potential as fillers in MMMs, owing to their large specific surface area, high aspect ratio, layered structure, and nanoscale thickness.^48^ For instance, Qin et al.^76^ synthesized Cu-MOF nanosheets with a thickness of 20 nm and incorporated them into a Pebax 3533 matrix to produce MMMs for CO_2_/N_2_ separation. The prepared MMMs containing 0.2 wt % Cu-MOF exhibited a high CO_2_ permeability of 619 Barrer and an ideal CO_2_/N_2_ selectivity of 24. It was found that the Cu-MOF nanosheets could form a unique stacked configuration in a polymer matrix, which facilitates CO_2_ transport but inhibits N_2_ transport by forming more tortuous pathways. The low loading of fillers and the improved interfacial compatibility owing to the high aspect ratio of Cu-MOF ensured the homogeneous dispersion of Cu-MOF in MMMs. As the above studies show, the modification of MOFs not only effectively tunes their structural properties, such as BET surface area, pore volume and pore size, and physicochemical properties such as CO_2_ affinity, functionality groups, and hydrophobicity, but also improves their compatibility with the polymer matrix to mitigate the formation of interfacial defects and the agglomeration of filler. As a result, the CO_2_/N_2_ separation performance can be enhanced, and the long-term stability of MMMs can be ensured.

COFs are porous materials covalently linked by organic monomers, and have been used as fillers in MMMs owing to their superior compatibility with polymer matrices and their ordered and modifiable pore structures.^77^ For instance, Chang et al.^45^ modified TPB-DMTP-COF with [Emim]­[Tf_2_N] IL to enhance its CO_2_ affinity and to narrow its pore size. The modified IL@COF was incorporated into PIM-1 to prepare MMMs for CO_2_/N_2_ separation. The 3 wt % IL@COF/PIM-1 MMMs exhibited a CO_2_ permeability of 9,138 Barrer and a CO_2_/N_2_ selectivity of 20, this being related to the enhanced CO_2_ affinity, improved size-sieving ability, and improved interfacial compatibility. What is more, the prepared MMMs demonstrated superior antiplasticization capabilities in combination with long-term separation stability. Dai et al.^46^ fabricated β-ketoenamine-linked TpTta-COF/PIM-1 MMMs for CO_2_/N_2_ separation. MMMs containing 6 wt % COF achieved a CO_2_ permeability of 9672 Barrer and a CO_2_/N_2_ selectivity of 26.3, owing to the strong CO_2_ affinity of the prepared COF, the high interfacial compatibility, and the formation of a continuous channel for CO_2_ molecules. As the foregoing examples show, PIMs^46,66,78^ are preferred for use as polymer matrices in MMM preparation, owing to their high gas permeability. The incorporation of fillers such as COFs into PIMs increases their CO_2_ permeability and CO_2_/N_2_ selectivity, and enhances their antiplasticization and antiaging abilities.^45^

### 5.3 Inorganic Membranes

Porous inorganic membranes, such as zeolite membranes,^85,86^ and MOF and COF membranes^87–89^ are fabricated for use in CO_2_/N_2_ separation, owing to advantages such as large surface area, high porosity, tunable pore size and surface properties, highly stable thermal and chemical stability, and ease of modification and functionalization. Recent developments in inorganic membranes for CO_2_/N_2_ separation are summarized in Table 4. Inorganic membranes have demonstrated high CO_2_ permeance and moderate CO_2_/N_2_ selectivity. Zeolite membranes are desirable candidates for CO_2_ separation due to their ordered molecular-level pores and tunable surface properties. Co-exchanged SSZ-13 zeolite membrane was produced via the seeded growth of Na-SSZ-13 on a porous Al_2_O_3_ support and the subsequent ion exchange of Co^2+^ and Ca^2+^. The morphology and structure of the 4.4 μm continuous SSZ-13 membranes were well preserved after the ion exchange treatment. The prepared Co-SSZ-13 zeolite membrane exhibited the highest CO_2_ permeance (198 GPU) and CO_2_/N_2_ selectivity (28), owing to its reduced pore size and enhanced electrostatic and coordination interactions with CO_2_ molecules.^85^

**Table 4:** Representative Examples of Recently Developed (in the Last Five Years) Inorganic Membranes for CO_2_/N_2_ Separation (1 GPU = 3.35 × 10^–10^ mol m^–2^ s^–1^ Pa^–1^, 1 Barrer = 3.35 × 10^–16^ mol m m^–2^ s^–1^ Pa^–1^)

membrane materials	fabrication method	operating conditions	CO_2_/N_2_ mixture	CO_2_ permeability/permeance	CO_2_/N_2_ selectivity	long-term performance	refs
NH_2_-MIL-101(Cr)/α-Al_2_O_3_ substrate	In situ growth	20 °C, 1.1 bar	15/85 vol%	6104 GPU	37	Stable separation performance for 50 h	^87^
Tungsten-doped MFI zeolite/α-Al_2_O_3_ substrate	Dip coating; in situ growth	25 °C, 1 bar	50/50 mol %	507 GPU	29	Stable separation performance for 180 h	^95^
Co-SSZ-13 zeolite/Al_2_O_3_ disc	In situ growth	25 °C, 3 bar	50/50 vol%	198 GPU	27.7	Stable separation performance for 70 h	^85^
SAPO-34/Al_2_O_3_ tube	Secondary growth	25 °C, 1 bar	15/85 vol%	5430 GPU	32.9	Stable separation performance for 120 h	^86^
PDA@ZIF-L/PSf	In situ growth	30 °C, 1 bar	15/85 vol%	1200 GPU	14.5	Stable separation performance for 200 h	^96^
MIL-101/a_g_Zn–P-dmbIm glass membrane	In situ melting	25 °C, 2 bar	50/50 vol%	18,670 Barrer	61	Stable separation performance for 120 h	^88^
ZIF-8 doped carbon molecular sieves (ZCMS)	Oxidation and carbonization processes	30 °C, 0.1 bar	50/50 mol %	8285 Barrer	24.1	Stable separation performance for 20 cycles of experiment	^97^
c-oriented MIL-125	Secondary growth	25 °C, 1 bar	15/85 vol%	720 GPU	47.7	Stable separation performance for 20 h	^92^
TpPa-SO_3_H COF/PAN	Vacuum-assisted self-assembly	25 °C, 2 bar	20/80 vol%	1371 GPU	33	The selectivity and CO_2_ permeance of the membrane decreased by 10.2% and 12.5% in the initial 32 h, afterward becoming stable	^89^
IL@TpPa-SO_3_H COF/PAN	Spin coating	25 °C, 2 bar	20/80 vol%	1116 GPU	20.3	Stable separation performance for 100 h	^90^
PEI-TpBD-(COOH)_2_ COF/PAN	Vacuum filtration	25 °C, 2 bar	20/80 vol%	1004 GPU	33.4	Stable separation performance for 100 h	^91^
MIL-140A/Al_2_O_3_ disc	In situ growth	30 °C, 1 bar	50/50 mol %	2571 GPU	59.8	Stable separation performance for 220 h	^93^
ZIF-62/anodic aluminum oxide	In situ growth	25 °C, 1 bar	50/50 vol%	540 GPU	31.4		^94^
MIL-125(Ti)/α-Al_2_O_3_ substrate	Dip coating; secondary growth	25 °C, 1 bar	15/85 vol%	127 Barrer	48.3	Stable separation performance for 20 h	^98^
Cu@NH_2_-MIL-125/α-Al_2_O_3_ substrate	In situ growth	25 °C, 1 bar	50/50 vol%	696 GPU	43.2	Stable separation performance for 20 h	^99^
UiO-66/α-Al_2_O_3_ substrate	Epitaxial growth	25 °C, 1 bar	15/85 vol%	2070 GPU	35.4		^100^

COFs are emerging materials for the preparation of membranes for gas separation, owing to their high porosity, large specific surface area, high thermal and chemical stability, adjustable geometry of molecular building blocks, and modifiable functional groups.^90,91^ Liu et al.^90^ synthesized TpPa-SO_3_H COF nanosheets via interfacial polymerization from 1,3,5-triformylphloroglucinol (Tp) and 2,5-diaminobenzenesulfonic acid (Pa-SO_3_H). Subsequently, the ionic liquid 1-butyl-3-methylimidazolium tetrafluoroborate ([BMIM]­[BF_4_]) and COF nanosheets were physically mixed in ethanol/water solvent to form a casting solution. The [BMIM]­[BF_4_]@COF membranes were prepared by spin coating on a PAN support. It was found that the ionic liquids repaired the intramembrane defects and enhanced the CO_2_ solubility of the membrane. As a result, the prepared membranes achieved a high CO_2_ permeance of 1,116 GPU and CO_2_/N_2_ selectivity of 20.

MOF membranes with high CO_2_ permeability and CO_2_/N_2_ selectivity can be obtained by adjusting the pore structure, controlling the membrane thickness and framework orientation, and tailoring the surface functionality.^92,93^ Highly *c*-oriented defect-rich MIL-125 membranes were fabricated by combining a Ti-oxo cluster source with heterogeneous metal ion doping (Figure 6). The prepared MIL-125 membrane achieved a high CO_2_ permeance of 720 GPU and a CO_2_/N_2_ separation factor of 48 in separation tests using a CO_2_/N_2_ (15/75 vol %) gas mixture, and exhibited high performance stability for 20 h.^92^ Yan et al.^93^ prepared a (200)-oriented MIL-140A membrane with 55 nm thickness using l-histidine-modulated MIL-140A nanosheets as a seed layer, via epitaxial growth of MIL-140A membrane. The prepared MIL-140A membrane achieved a high CO_2_ permeance of 2,572 GPU and a CO_2_/N_2_ separation factor of 60 in separation tests on an equimolar CO_2_/N_2_ gas mixture. It was found that the l-histidine modulator could regulate the microstructure of the membrane, control the pore size and configuration to improve the size-sieving effect, and provide amino groups serving as CO_2_ carriers.

**Figure 6:**
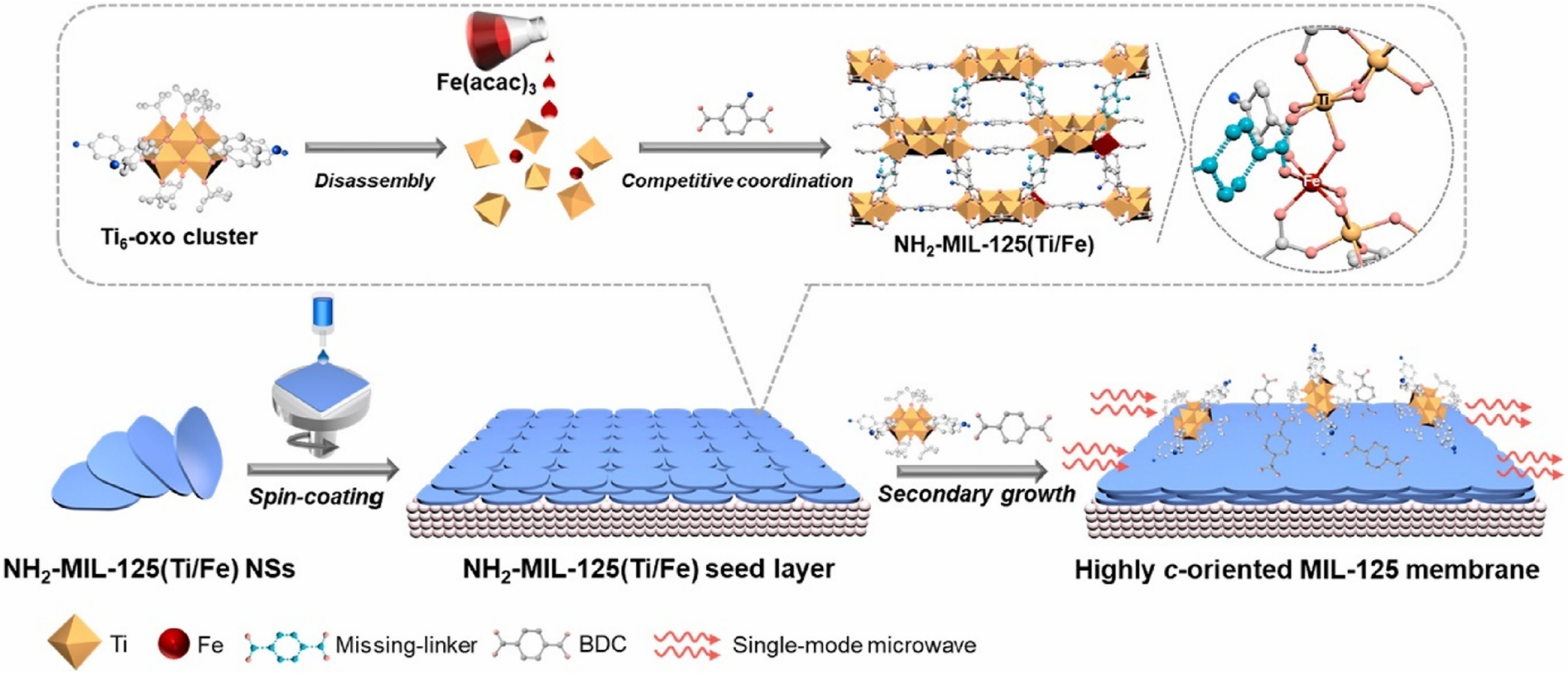
Schematic diagram of the fabrication of highly c-oriented defect-rich MIL-125 membrane.^92^ Reprinted with permission from Elsevier. Copyright 2024.

As shown by the above examples and Table 4, inorganic membranes are generally fabricated on porous supports. The CO_2_ permeance and CO_2_/N_2_ selectivity of inorganic membranes can be increased by reducing the membrane thickness, precisely controlling the pore size, and modifying the surface properties. Inorganic membranes can potentially be used in a process of direct CO_2_ capture from air, due to their high CO_2_ separation performance. However, the high production cost and difficulty in upscaling limit their large-scale applications. This is because the formation of defect-free zeolite and MOF membranes via crystal growth is challenged by the stringent requirements concerning the reaction conditions, such as the control of nucleation, crystal growth, and crystal orientation.^93,94^

The CO_2_ separation performances of polymeric membranes, MMMs, and inorganic membranes, summarized in Tables 2–4, are presented and compared in Figure 7. It can be seen from Figure 7a that free-standing MMMs surpass the Robeson upper bound and demonstrate better CO_2_ permeability and CO_2_/N_2_ selectivity than free-standing polymeric membranes. This is because the incorporation of nanofillers such as MOFs, COFs and GO can effectively adjust the transport pathways for CO_2_ molecules. For example, the incorporated porous MOFs and COFs in MMMs can provide additional transport pathways for CO_2_ molecules while inhibiting the transport of N_2_ molecules, through tuning of their porosity and pore size. Incorporated GO nanosheets in MMMs can create selective and permeable nanochannels for the faster transport of CO_2_ molecules, but more distorted diffusion pathways for larger N_2_ molecules, resulting in higher diffusion resistance. What is more, the modification of nanofillers with CO_2_-philic groups can further enhance their compatibility with the polymer matrix and increase CO_2_ solubility in MMMs. The CO_2_ separation performance of free-standing polymeric membranes is limited by the trade-off relationship and the high membrane thickness. Therefore, compared with free-standing polymeric membranes, free-standing MMMs offer greater potential for use in m-DAC processes.

**Figure 7:**
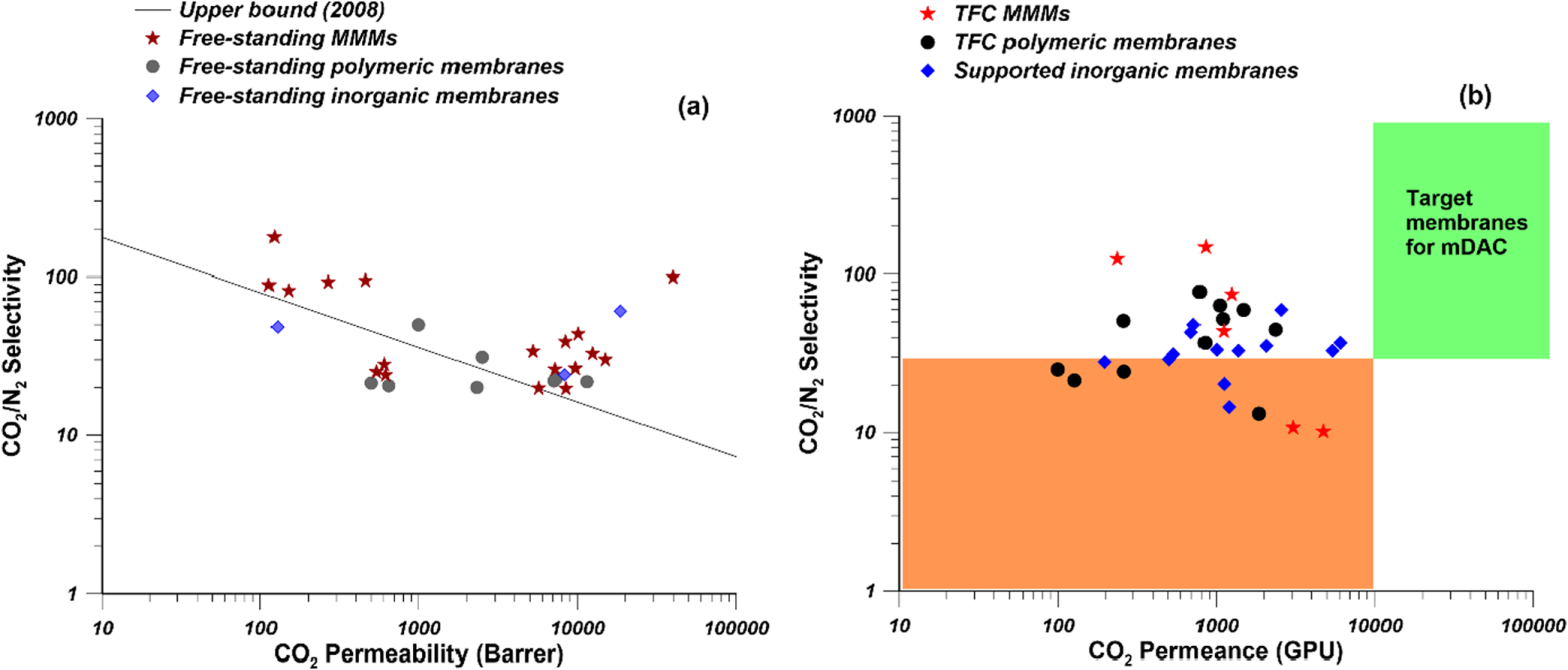
Comparison of (a) free-standing and (b) thin film composite (TFC) polymeric membranes, MMMs, and inorganic membranes for CO_2_ separation performance (data from Tables 2–4).

As shown in Figure 7b, the CO_2_ separation performances of TFC polymeric membranes and supported inorganic membranes are comparable; they exhibited high CO_2_ permeance and moderate CO_2_/N_2_ selectivity. Owing to the high production cost and difficulty of mass production of defect-free supported inorganic membranes, the fabrication of TFC polymeric membranes is more cost-effective, and these are more promising for large-scale application in m-DAC processes. The TFC MMMs exhibited both high CO_2_ permeance and high CO_2_/N_2_ selectivity, owing to a combination of the advantages of nanofillers and the polymer matrix. The fabrication of TFC MMMs with high CO_2_ capture performance may be an advantageous way to achieve target membranes for m-DAC processes.

## 6 m-DAC Processes for CO_2_ Mitigation

The development of m-DAC processes is essential for lowering greenhouse gas emissions and reducing CO_2_ concentration in the atmosphere, and m-DAC is considered a negative emission technology. Nevertheless, direct CO_2_ capture using m-DAC is challenging, owing to the low CO_2_ concentration in the air. Reports on applications of membranes in the m-DAC process are less common in the literature than those on applications of membranes in CO_2_ capture from point-source emissions such as flue gas. In this section, the recent applications of membranes for m-DAC are presented and discussed.

The high CO_2_ permeance and CO_2_/N_2_ selectivity of membranes are crucial for their use in the DAC process, owing to the low partial pressure of CO_2_ in the atmosphere. Facilitated transport membranes exhibit high CO_2_ separation performance exceeding the upper bound, since CO_2_ transport through such membranes is not limited by the solution–diffusion mechanism. A 900 nm thin film composite facilitated transport membrane, PIL-IL/GO, was developed by casting a solution containing IL, PIL and GO on a PES/PET support, and was used for CO_2_ capture from atmospheric air and cabin air.^101^ Owing to the highly efficient facilitated transport mechanism, the prepared PIL-IL/GO membrane achieved a high CO_2_ permeance of 3,092 GPU and CO_2_/N_2_ selectivity of 1,189 in the case of CO_2_ capture from atmospheric air (410 ppm of CO_2_ feed, 295 K and 40% relative humidity), and a CO_2_ permeance of 620 GPU and CO_2_/N_2_ selectivity of 257 in the case of CO_2_ capture from cabin air (2500 ppm of CO_2_ feed, 295 K and 40% RH). Moreover, the membrane demonstrated high stability over a two-week period of continuous separation, owing to the intermolecular interactions among the PIL, IL, and GO components of the selective layer over the poly­(ethersulfone)/poly­(ethylene terephthalate) (PES/PET) substrate.^101^ It can be concluded that the prepared PIL-IL/GO membrane has demonstrated high potential for application in m-DAC processes and for the removal of CO_2_ from cabin air in spacecraft and buildings. Lee et al.^102^ developed another PIL-IL/GO thin (850 nm) film composite facilitated transport membrane, containing [EMIM]­[2-CNpyr] as mobile carrier and P­[DADMA]­[2-CNpyr] as fixed carrier, on a highly permeable bicontinuous structured poly­(ethersulfone)/poly­(ethylene terephthalate) (bPES/PET) substrate. The prepared membrane exhibited a high CO_2_ permeance of 2,100 GPU, CO_2_/N_2_ selectivity of 1,100, and CO_2_/O_2_ selectivity of 265 under the conditions 410 ppm of CO_2_, 40% RH, and 295 K, which demonstrated the possible application of such membranes in DAC processes. Moreover, the prepared membranes displayed high separation stability over 7 days’ continuous testing. Thus, owing to the nanoconfinement of PIL-IL within the GO layer through ionic interactions between the carriers and the GO flakes and π–π interactions between the aromatic moieties, the stability of the membrane was improved, facilitating its long-term use. Aiming to produce a membrane for use in an m-DAC process, Kamio et al.^103^ developed a hollow fiber facilitated transport membrane via the deposition of poly­(vinylethylimidazolium glycinate) (poly­([Veim]­[Gly])) gel particles on the inner surface of a polysulfone (PSf) hollow fiber support. The prepared membrane exhibited a high CO_2_ permeance of 1,400 GPU and a CO_2_/N_2_ selectivity of 2000 at 30 °C under atmospheric pressure with a CO_2_ partial pressure of 0.1 kPa, owing to the facilitated transport mechanism for CO_2_ molecules.^103^ In our opinion, the prepared hollow fiber membrane is suitable for DAC applications, due to its high CO_2_ separation performance at low CO_2_ partial pressure and the ability to produce a compact membrane module with high specific surface area and high volume efficiency, which also demonstrates the importance of detailed membrane design.

As discussed above, thin film facilitated transport membranes containing mobile CO_2_ carriers (ILs and amino acids) and fixed CO_2_ carriers (PILs) are fabricated on a porous polymeric support, and they demonstrate high CO_2_ capture performance at low CO_2_ concentrations. To further enable the application of thin film facilitated membranes in m-DAC processes, key tasks include the utilization of hollow fiber support for the production of the compact membrane module, the development of techniques for the formation of a thinner selective layer to achieve high gas permeance, and optimization of the porous structure of the membrane support to minimize the gas transport resistance.

Adsorptive membranes have demonstrated high effectiveness in direct CO_2_ capture from air, owing to the interactions between CO_2_ molecules and the abundant CO_2_-philic functional groups.^104–106^ He et al.^104^ fabricated polyethyleneimine-grafted poly­(vinylidene fluoride) (PVDF-g-PEI) hollow fiber membranes for m-DAC using the dip-coating and cross-linking methods. Hollow fiber membranes possess high surface area-to-volume ratios, as well as good flexibility in upscaling. The highly porous and honeycomb-like structure of hollow fiber membranes facilitates CO_2_ adsorption at low concentrations, owing to the high surface area with CO_2_-philic groups accessible to the CO_2_ in air. A fabricated membrane containing 6.6 wt % PEI attained the highest CO_2_ adsorption capacity of 0.5 mmol CO_2_ per 1 g of membrane. The prepared membranes exhibited a 3.33% loss of capacity and 97% performance regeneration over 20 cyclic CO_2_ adsorption–desorption experiments, demonstrating their long-term stability. Summarizing, PVDF-g-PEI hollow fiber membranes also display high scalability for large-scale m-DAC applications. Zhang et al.^105^ prepared PAN–PEI-ECH hollow fiber membranes via interfacial polymerization between polyethyleneimine (PEI) and epichlorohydrin (ECH) on the inner surface of polyacrylonitrile (PAN) hollow fibers. Subsequently, the prepared membranes were used in DAC of CO_2_ under ambient conditions (420 ppm, 20–25 °C, RH = 30–50%). The fabricated PAN–PEI-ECH hollow fiber membranes exhibited high CO_2_ adsorption capacity of 2.35 mmol CO_2_ per 1 g of membrane and high adsorption stability after 18 cycles of adsorption and desorption experiments. Hence, it can be concluded that these membranes have high potential in capturing CO_2_ at low concentrations. As the above examples show, adsorptive hollow fiber membranes demonstrate good scale-up ability for applications in DAC processes, owing to their high effectiveness and stability in CO_2_ capture at low concentrations. For adsorptive membranes, it is important to generate CO_2_-philic functional groups acting as CO_2_ adsorption sites on the hollow fiber support. Moreover, the regeneration of CO_2_ adsorption sites must be highly efficient in repetitive CO_2_ adsorption–desorption experiments to guarantee the long-term stability of adsorptive membranes. The features and advantages of facilitated transport membranes and adsorptive membranes, as discussed above, are summarized in Figure 8. Both facilitated transport membranes and adsorptive membranes demonstrate high potential in large-scale applications in m-DAC processes.

**Figure 8:**
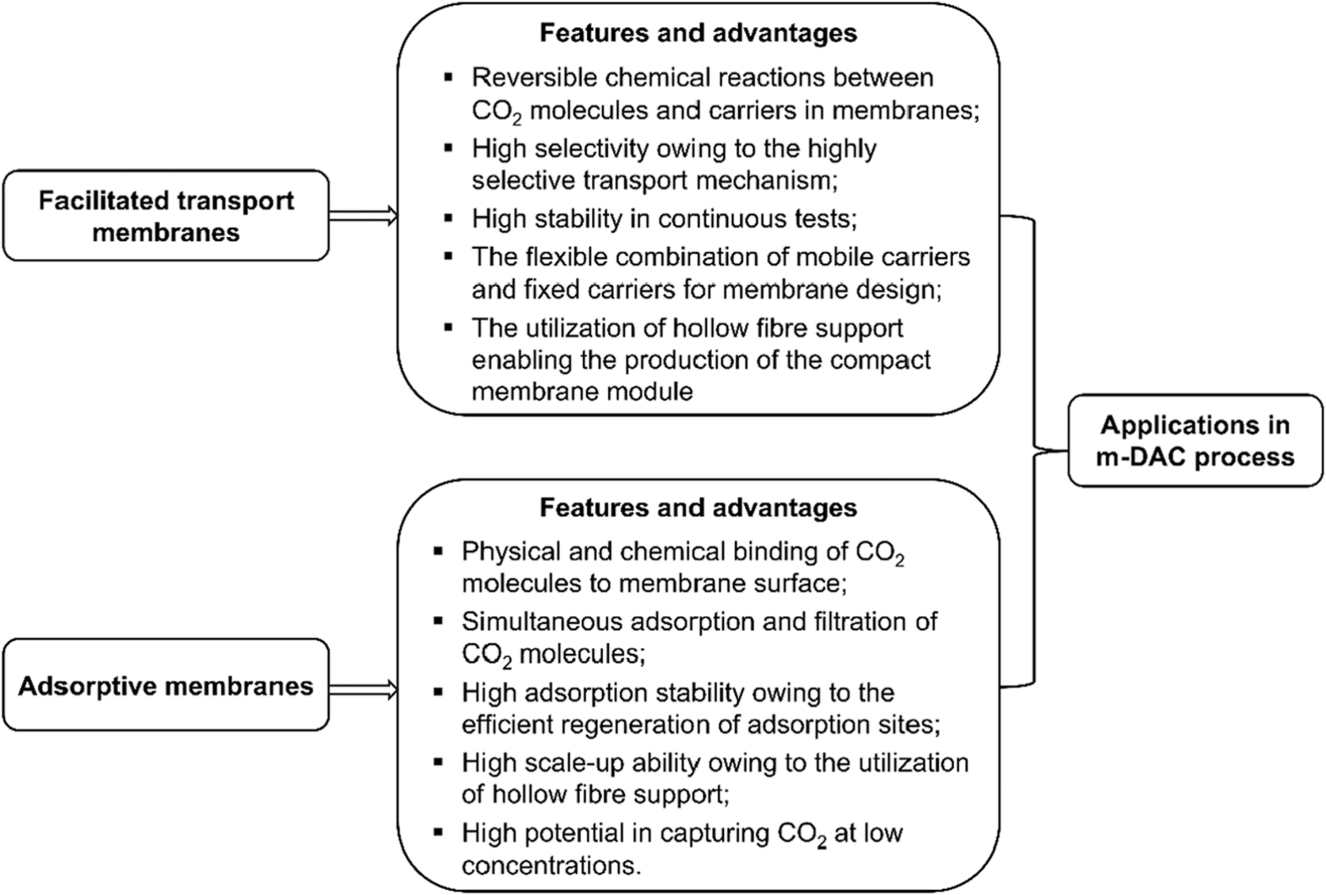
Summary and comparison of facilitated transport membranes and adsorptive membranes in m-DAC processes.

## 7 Current Challenges and Future Perspectives

The m-DAC process is highlighted as a promising carbon negative technology to mitigate climate change. The main challenges for the large-scale implementation of the process lie in the design of membrane systems and the fabrication of high-performance membranes for capturing low-concentration CO_2_ from air. The large-scale implementation of m-DAC is still in its early stages. This is because the efficient capture of low concentrations of CO_2_ from air is associated with high energy consumption and considerable thermodynamic requirements, resulting in the high cost of m-DAC processes. To tackle these challenges, a multistage membrane separation process is recommended for m-DAC, since it can increase the purity of the captured CO_2_ and reduce the energy consumed in capturing 1 kg CO_2_. In addition, the selection of a membrane with CO_2_/N_2_ selectivity higher than 30, and the attainment of a high pressure ratio and low stage cut can ensure higher CO_2_ purity and a reduction in energy consumption. A techno-economic assessment has shown the cost of an m-DAC process to be between $91/tCO_2_ and $95/tCO_2_, which is commercially viable, subject to the conditions of lower oil prices and appropriate policy support.^33^

The design of novel hybrid separation systems, such as the integration of m-DAC with a sorption-based DAC process, and the utilization of membrane contactors,^107^ is recommended to achieve efficient CO_2_ capture from air. Last but not least, the m-DAC process should be combined with a CO_2_ utilization process, such as the conversion of CO_2_ into fuels or valuable chemicals, to address energy security and climate change issues. The key challenge to the combined m-DAC system with CO_2_ reduction is the presence of impurity gases such as oxygen (O_2_). The presence of O_2_ is disruptive to the CO_2_ reduction reaction since O_2_ can be preferentially reduced. Therefore, it is of great importance to endow membranes with a high CO_2_/O_2_ selectivity to enable their application in such a combined system.^108^ In the literature, most studies focus on the separation of CO_2_ from N_2_, CH_4_ or H_2_. Therefore, more attention should be paid to CO_2_/O_2_ separation while designing membranes for m-DAC processes. Additionally, more research should focus on the scaling up of m-DAC processes to industrial levels, tackling the challenges of energy requirements and infrastructure. Research should be carried out on a cleaning strategy for membranes used in practical m-DAC processes, since the air throughput of the membranes is extremely high, and the particulate matter present in air might impair the membrane’s performance. Cooperation between academia, industry, and policymakers may serve to facilitate the implementation of large-scale m-DAC process.

It should also be highlighted that membranes with high CO_2_ permeance and CO_2_/N_2_ selectivity are crucial for efficient CO_2_ capture in DAC processes. The trade-off relationship between permeance and selectivity, plasticization and physical aging are the main challenges for the fabrication of high-performance polymeric membranes. Recently developed polymeric membranes, MMMs, and inorganic membranes for CO_2_/N_2_ separation have shown high potential for application in m-DAC processes, owing to their enhanced CO_2_ separation performance. However, more research is needed to fabricate membranes with high CO_2_ permeance to meet the criteria (CO_2_ permeance >10,000 GPU and CO_2_/N_2_ selectivity >30) for m-DAC processes defined by Fujikawa et al.^26^ To achieve this goal, the following four directions are proposed. First, reducing the membrane thickness to less than 1 μm may dramatically increase CO_2_ permeance. Therefore, the development of thin film formation techniques is strongly needed in order to obtain defect-free thin membranes with high gas permeance and selectivity. The utilization of commercially available rubbery polymers such as Pebax and PDMS, and ecofriendly chemicals and solvents such as PEG, water, and ethanol may further enable the mass production of TFC membranes and their large-scale application in CO_2_ capture processes. Second, more research needs to be done on the development and utilization of high-performance polymers for membrane preparation. Novel polymers, such as CO_2_-philic polymers and polymers with high microporosity, should be developed for the preparation of membranes with high CO_2_ separation performance. For glassy polymeric membranes, modifications of polymers—such as the introduction of CO_2_-philic groups or rigid bulky units into the polymer structure—and additional treatments such as cross-linking have the potential not only to enhance the CO_2_/N_2_ separation performance, but also to improve the antiplasticization and antiaging abilities of membranes. For example, PIMs can be synthesized and functionalized via copolymerization, chemical modification and thermal cross-linking. The resulting PIM membranes have displayed not only high CO_2_ permeance and CO_2_/N_2_ selectivity, owing to their designed high pore volume and enhanced CO_2_ affinity, but also antiaging and antiplasticization abilities. Third, the utilization of nanofillers such as MOFs, COFs, and 2D materials in MMMs has enhanced their CO_2_ separation performance through manipulation of the free pore volume and CO_2_ affinity of membranes, as well as gas molecule transport pathways. Moreover, the antiaging and antiplasticization abilities of membranes are enhanced by the interactions between nanofillers and polymer chains. The functionalization of nanofillers is highly recommended to enhance their compatibility with the polymer matrix and achieve the homogeneous distribution of nanofillers in the matrix. Lastly, facilitated transport membranes have demonstrated both high CO_2_ permeance and high CO_2_/N_2_ selectivity, owing to their facilitated transport mechanism for CO_2_ molecules. To date, however, the application of facilitated transport membranes in m-DAC processes has been studied only on a laboratory scale. Finally, the stability of mobile carriers in membranes must be enhanced when preparing facilitated transport membranes, to guarantee their high CO_2_ separation performance.^16^ The current status, challenges, and a roadmap for the future development and the application of m-DAC processes, as discussed above, are summarized and presented in Figure 9.

**Figure 9:**
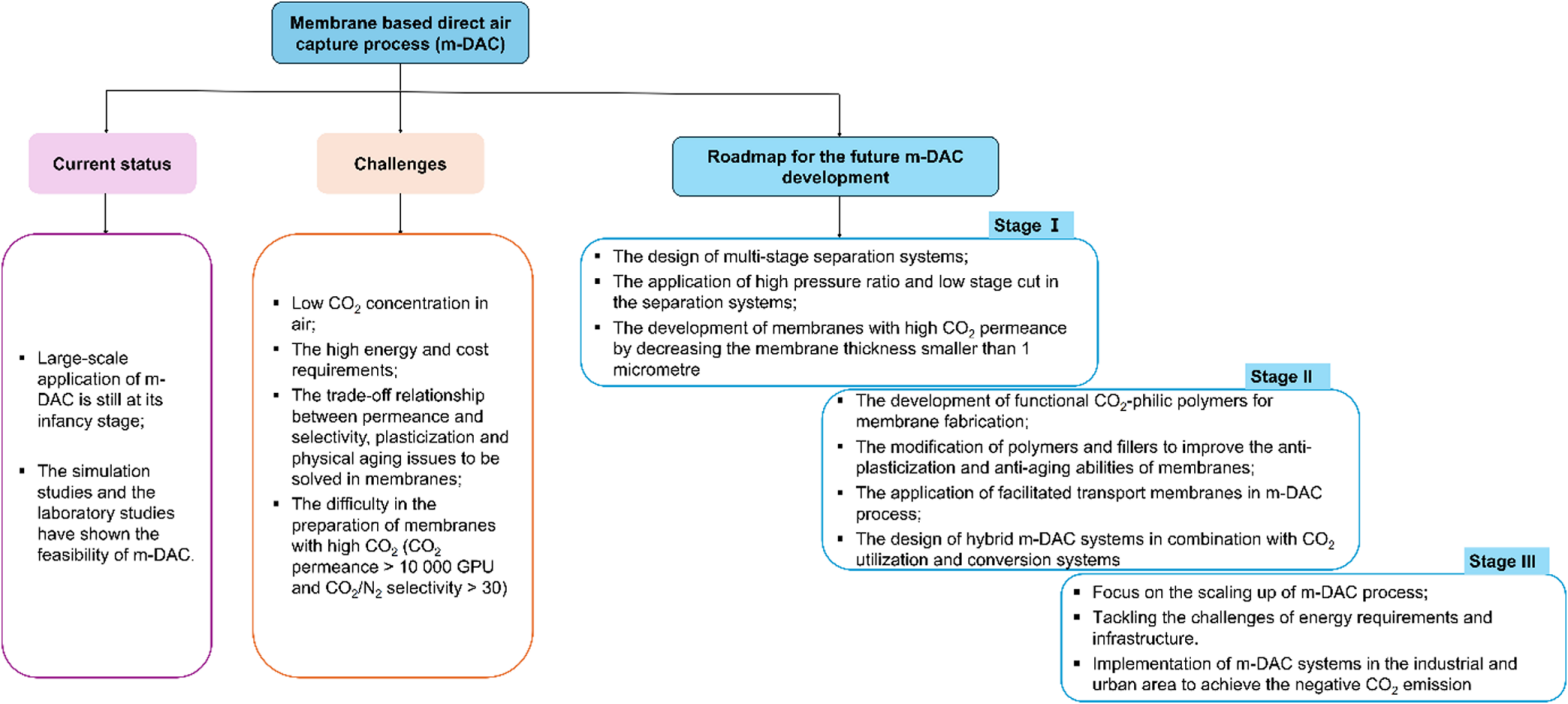
Summary of the current status, challenges, and roadmap for the future development for the m-DAC process.

## 8 Conclusions

Alongside the CCS process for the reduction of CO_2_ emissions, the DAC process, as a carbon negative technology, is a complementary approach to tackling climate change and limiting the rise in temperatures. Nevertheless, it is challenging to apply membranes in DAC processes, due to the low CO_2_ concentration in air. The m-DAC process has several advantages over the sorption-based DAC process, including higher energy efficiency, simple operation, high scale-up ability, high packing density and modularity, and small carbon footprint.

Owing to the low CO_2_ concentration in air and the high gas throughput of the m-DAC process, the membranes used in this process are required to offer high CO_2_ permeance and CO_2_/N_2_ selectivity. In this review, recently developed membranes, including polymeric membranes, MMMs, and inorganic membranes for CO_2_/N_2_ separation, have been summarized and analyzed to highlight their potential applications in m-DAC. The inorganic membranes demonstrated high gas permeance and CO_2_/N_2_ selectivity. However, their high production cost and difficulties in upscaling limit their large-scale application. More research is needed to study the effect of humidity and impurities on their long-term stability. Compared with inorganic membranes, polymeric membranes and MMMs are more feasible for m-DAC applications, owing to their lower cost of manufacture and high processability. The development of novel thin film formation techniques is crucial for the fabrication of defect-free thin film composite membranes with thicknesses on the scale of a few hundred nanometers. Thin film composite membranes are more suitable for the m-DAC process. In addition to high CO_2_ permeance and CO_2_/N_2_ selectivity, membranes used in the m-DAC process should possess high antiplasticization and antiaging abilities to guarantee their long-term performance stability. Lastly, novel polymers such as CO_2_-philic polymers and polymers with high microporosity should be developed for the preparation of membranes with good CO_2_ separation performance. The combination of the m-DAC process with carbon utilization and storage has great potential to establish a future sustainable and circular economy enabled by carbon recycling and negative carbon emissions.

## List of Acronyms and Abbreviations

CCS: carbon capture and sequestration
COFs: covalent organic frameworks
DAC: direct air capture
GO: graphene oxide
GPU: gas permeance unit, 1 GPU = 3.35 × 10^–10^ mol m^–2^ s^–1^ Pa^–1^
IPCC: International Panel on Climate Change
m-DAC: membrane-based direct air capture
MMMs: mixed matrix membranes
MOFs: metal–organic frameworks
PDMS: poly­(dimethylsiloxane)
PEG: poly­(ethylene glycol)
PEO: poly­(ethylene oxide)
PI: polyimide
PIMs: polymers of intrinsic microporosity
PSf: polysulfone
PTMSP: poly­[1-(trimethylsilyl-1-propyne)]
SBI: spirobisindane
TF: transient-filler treatment
TFC: thin film composite
φ: pressure ratio
θ: stage cut
